# Community Trait Variation Drives Selection on Species Diversity Through Feedback With Predator Density

**DOI:** 10.1002/ece3.70477

**Published:** 2024-10-23

**Authors:** Phuong‐Anh Vu, Lutz Becks

**Affiliations:** ^1^ Aquatic Ecology and Evolution University of Konstanz Konstanz Germany

**Keywords:** community assembly, community dynamics, ecological selection, predator–prey, trait distribution

## Abstract

Identifying the processes underlying community assembly and dynamics remains a central goal in ecology. Although much research has been devoted to analyzing how environments affect species diversity, fewer studies have resolved the link between the fundamental process of ecological selection and species diversity. It has been suggested that identifying ecological selection by estimating changes in community‐weighted variance (CWV) and mean (CWM) of functional traits may help to identify more general rules of community assembly. Here, we asked whether and how selection by predation and competition affect species diversity, and how this is determined by the initial CWV and CWM for traits governing species interactions, as in our case: Competitiveness and defense against a predator. We tracked experimental five‐species phytoplankton communities in the presence and absence of a rotifer predator over time. We manipulated the initial community composition so that communities shared at least three of the five species but differed in CWV and CWM for defense against predation. We found that species diversity was highest with higher initial trait distributions and that temporal changes in diversity correlated with trait selection. The initial distributions determined the form of selection over time, with directional selection for defense and competitiveness, followed by reduced selection and an increase in niche availability when the initial trait distribution was low or high. For intermediate initial trait distributions, we observed directional selection in only one trait, followed by stabilizing selection. Differences and changes in selection for defense, competitiveness, and species diversity correlated with the changes in predator density over time. This suggests that the initial trait distribution determined species diversity through a feedback loop with changes in selection on traits and predator density. Overall, our study shows that identifying ecological selection on functional traits can provide a mechanistic understanding of community assembly.

## Graphical abstract

Our work addresses a fundamental problem in ecology: how to explain community assembly. We apply a recently proposed approach to link species diversity and trait variations within communities via patterns of selection. By tracking selection, diversity, trait variations, and population dynamics in experimental phytoplankton communities that differ in initial trait and species composition, we provide the first paired observations of species diversity, trait change, and population dynamics, and discuss how these traits respond and drive each other in a feedback loop.

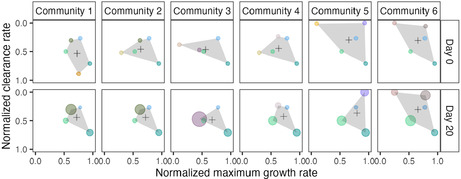

## 1 Introduction

A long‐standing question in community ecology is how very large numbers of species can persist (Hutchinson 1959; Mora et al. 2011) and why the number and distribution of species present varies across habitats (Brown 2014; Hillebrand 2004; MacArthur, Diamond, and Karr 1972). Species interactions, such as competition and predation, are important drivers of the local species pool in addition to abiotic conditions and may act altogether with other community filters at larger geographic and temporal scales (Götzenberger et al. 2012; Keddy 1992; Kraft et al. 2015; Mittelbach and Schemske 2015). However, disentangling the underlying mechanisms of species interactions for community assembly and dynamics remains a major challenge.

A number of general rules of species interactions for community assembly have been identified (e.g., the role of limited resources (Tilman, Kilham, and Kilham 1982), differences in niche and competitive ability (Chesson 2000), or predation (Chesson and Kuang 2008)). For a long time, much of the existing research was dominated by the use of taxonomic identities of species and measures of species diversity, i.e., richness or evenness, to study community assembly and dynamics (Diamond 1973; MacArthur and Wilson 1963; Williams 1947). Over the last decades, the interest in trait‐based approaches has extensively increased (Ackerly and Cornwell 2007; Kraft, Godoy, and Levine 2015; Lajoie and Kembel 2019; Litchman and Klausmeier 2008; Weithoff and Beisner 2019; Westoby and Wright 2006). This approach uses functional traits, where a trait is a well‐defined, measurable property at the individual level, and species within a community can be grouped based on their traits (McGill et al. 2006). Traits provide a common and taxon‐independent currency, and the functional trait composition of a community should reflect the outcome of key assembly rules, including those based on biotic interactions (Spasojevic and Suding 2012). Therefore, trait‐based approaches have the potential to reveal generalizable patterns across environments (Harley 2011; Harmon et al. 2009; Violle et al. 2007). The influence of traits on community assembly and dynamics is, however, highly context‐dependent, not least because trait changes and population dynamics can interact in a feedback loop.

Ecological selection is the result of deterministic fitness advantages between individuals of different species resulting from the biotic and abiotic filtering of species (DeMalach, Ke, and Fukami 2022). It favors certain trait combinations carried by species, changing the composition of communities (Vellend 2010, 2016). Therefore, identifying ecological selection on functional traits, i.e., traits with a strong influence on the performance of the organism in its environment (McGill et al. 2006) and that determine species interactions could improve a mechanistic understanding of community assembly and link the patterns of species diversity and trait variation within communities (DeMalach, Ke, and Fukami 2022; Rolhauser and Pucheta 2017; Vellend 2010, 2016). Within here, we refer to *selection* when speaking about *ecological selection*. DeMalach, Ke, and Fukami (2022) proposed a framework for studying the link between processes and patterns: They predict that directional or stabilizing selection (*sensu* Vellend 2016) on the community will result in lower species diversity. While both types of selection should decrease the community‐weighted variance (CWV) of the trait, there should be no change for stabilizing selection in the community‐weighted mean (CWM) of the trait but shifts in CWM for directional selection. This framework can also be used to address niche availability in communities, as niche theory predicts that limiting similarity within a community leads to divergence in functional traits related to resource use, defense mechanisms against consumers, and other processes that regulate population growth (MacArthur and Levins 1967; Stubbs and Bastow Wilson 2004).

This framework has been used to explain community composition across environments (DeMalach, Ke, and Fukami 2022), but it has rarely been tested in communities that initially differ in trait distribution, species composition, and that are under predation pressure. A higher community trait variation can lead to more available niches and thus selection for higher species diversity. However, larger differences in traits can also lead to a faster exclusion of species as selection strength increases with larger fitness differences (Vellend 2016). Therefore, differences in initial trait distribution may change selection and result in different outcomes in community structures and population dynamics. Another important source of selection shaping community assembly and dynamics is predation. It can promote species diversity through various mechanisms, e.g., oscillating population dynamics (Huisman and Weissing 1999; Vandermeer 2006), different types of species interactions (Mougi and Kondoh 2012), the interaction of competition and predation (Becks and Arndt 2008; Chesson and Kuang 2008; Meyer and Kassen 2007), or contemporary evolution. Applying the framework to shorter timescales and in conditions where selection on traits dynamically drives and is driven by population size changes, such as eco‐evolutionary feedback dynamics (De Meester et al. 2019; Fussmann, Loreau, and Abrams 2007; Hendry 2017) in predator–prey systems (Becks et al. 2010; Klauschies, Vasseur, and Gaedke 2016; Yoshida et al. 2003), can help to understand how selection determines community assembly (Chesson 2000) by linking species diversity, trait variation, niches, and population dynamics.

In this study, we tested the hypothesis that changes in species diversity over time are affected by selection imposed by predation and that this depended on initial trait distribution. Further, as predator–prey interactions are dynamic, this might also lead to changes in selection over time. More specifically, we used experimental phytoplankton communities and investigated how differences in initial trait distribution and the presence or absence of a predator, the rotifer *Brachionus calyciflorus*, affect species diversity (as evenness), community‐weighted trait distributions (variance and mean), and the population dynamics. We manipulated the phytoplankton communities' trait variation by composing communities of five species that differed in their competitiveness and defense against predation by the predator. To describe the competitiveness of the phytoplankton species, we used maximum growth rate as a trait, as it reflects their ability to efficiently acquire and utilize available resources, leading to faster reproduction, and potentially outcompete slower‐growing species (Edwards, Litchman, and Klausmeier 2013; Grover 1991; Litchman 2007). For defense, we measured predator clearance rate as it depicts the phytoplankton species ability to resist grazing of the predator (Pančić and Kiørboe 2018). Competition and defense have frequently been used to test trade‐off relationships in planktonic communities (Cadier et al. 2019; Ehrlich, Kath, and Gaedke 2020; Kasada, Yamamichi, and Yoshida 2014; Litchman 2007; Litchman and Klausmeier 2008; Litchman, Edwards, and Klausmeier 2015; Pančić and Kiørboe 2018; Yoshida et al. 2003), and a recent study showed that these two traits are good proxies for competitiveness and defense against predation (Réveillon and Becks 2024). We composed the six communities in a way that the five phytoplankton species of each community covered different parts of a defense–competition trait space, resulting in increasing area, and thus varied in their initial trait distributions. All communities had at least three out of five phytoplankton species in common, and the difference in trait distribution was mainly the result of increased defense (i.e., the presence of one or two species with higher defense). Thus, we manipulated the CWV and CWM for the defense trait across communities but kept the CWM and CWV for the competitiveness trait and species diversity similar across communities. We followed community dynamics with and without predators over 20 days. As the predator‐free communities were dominated by one species, we did not further analyze these communities.

For the communities with predators, we followed the framework proposed by DeMalach, Ke, and Fukami (2022): First, we measured diversity to account for whether selection on species had occurred. Here, we calculated diversity as evenness to attribute for the distribution of the species within communities. Second, we estimated CWV over time to determine whether selection on traits had taken place. Both indicated by a decrease in diversity and CWV between time points. Increases in CWV are interpreted as an increase in niche availability. Last, to identify the type of selection between time points, we calculated the CWM of the traits: If the decrease in CWV did not have an effect on CWM, selection was assumed to be stabilizing. However, if CWV decreased and CWM changed, selection was considered to be directional. We also analyzed how the temporal changes in phytoplankton community density correlated with changes in CWV and CWM of the defense and competitiveness over time, and how the differences in predator densities over time were driven by phytoplankton trait changes and densities. Finally, we discuss the potential feedback between species diversity, trait variation, and niche availability.

## 2 Materials and Methods

### 2.1 Species and Culture Conditions

We used 13 phytoplankton species *Acutodesmus obliquus* (formerly known as *Scenedesmus obliquus*), *Chlorella vulgaris*, *Monoraphidium minutum*, *Monoraphidium obtusum*, *Sphaerocystis* sp., *Chlamydomonas reinhardtii*, *Borodinellopsis texensis*, *Chloromonas augustae*, *Heterochlamydomonas rugosa*, *Lobochlamys culleus*, and two species of the genus *Cryptomonas* (Table S1). Species were chosen based on availability, their ability to grow under the same laboratory conditions, and being consumed by the rotifer predator. Prior to the experiments, all species were cultivated as monocultures in cell cultural flasks filled with 30 mL of sterile, nutrient repleted Woods Hole growth medium (1000 μmol NO_3_^−^ L^−1^, 65 μmol PO_4_^−^) at 20°C under continuous light (200 μmol photon m^−2^ s^−1^) and nonshaking conditions. As a predator, we used an asexually reproducing clone of the rotifer species *Brachionus calyciflorus*. The clonal line is derived from an isolate sampled in Milwaukee harbor (Bennett and Boraas 1989) which lost its ability to reproduce sexually (Fussmann et al. 2003). This allowed us to keep the predator traits constant over time. The rotifer stock cultures were maintained by weekly transfers to a sterile nutrient rich medium containing the green alga *M. minutum*. All experiments were conducted under the abovementioned culture conditions.

### 2.2 Trait Measurements

For the measurement of maximum growth rate as the estimate for competitiveness of the phytoplankton species, we inoculated 30 mL of sterile, nutrient‐rich growth medium with 1 × 10^3^ cells mL^−1^ and *n* = 3 replicates per species. We sampled 1 mL of each culture flasks daily for 12 days. All flasks were randomized every time after sampling to minimize the effect of spatial stochasticity. Samples were fixed (0.01% paraformaldehyde and 0.1% glutaraldehyde final concentration) and stored at 4°C in the dark before enumeration. Cell densities were measured from images that were acquired with an automated high‐content microscope (ImageXpress Micro 4, Molecular Devices, USA). For image acquisition, the samples were transferred to 96‐well plates with a flat bottom and stored for 24 h before imaging to allow for cells to settle to the bottom of the wells. Thirteen images per well were taken at 10× magnification using a Cy5 filter for detection of the chloroplasts' autofluorescence of the phytoplankton cells. Cells were identified and counted from the images using the MetaXpress analysis software with a customized analysis module. From the cell counts, we measured cell densities per day and estimated the growth curves of each replicate over a period of 12 days. From the mean densities of the replicates, we calculated the growth rate per day for each phytoplankton species using the following equation, where *N* is the population density (cells mL^−1^) and t is the time (day):

(1) $$\mathrm{GR}=\frac{\ln\left(N+1\right)-\ln\left(N\right)}{\left(t+1\right)-t}$$

We selected the maximum value for the maximum growth rate.

To obtain species trait values for defense against predation, we measured clearance rates of the predator for each of the phytoplankton species. For these assays, we centrifugated samples from the precultivated cultural flasks (3000 rpm for 10 min at 20°C) and resuspended the pellet in nitrate‐ and phosphate‐free medium to slow down algal growth during the assays for the clearance rates. We diluted the resuspended cultures to a density of 5 × 10^5^ cells mL^−1^ with the nitrate‐ and phosphate‐free medium and transferred 200 μL of each dilution into wells of 96‐well plates with *n* = 5 replicates for each phytoplankton species. We conducted the experiment in two treatments: One nongrazing, control treatment without the addition of predators to measure initial phytoplankton densities. For the grazing treatment, 1 day prior to the experiment, we filtered the stock culture of *B. calyciflorus* using a 40 μm nylon cell strainer to remove food algal cells from the culture and kept them in a nitrate‐ and phosphate‐free medium overnight. On the day of the experiment, we transferred five individuals of *B. calyciflorus* into each of the wells. After 8 h, we fixed cells by adding 5 μL of Lugol's iodine solution to each of the wells. The well plates were wrapped and stored at 4°C in the dark. Cell densities for both the control (*D*_initial_) and the grazing treatment (*D*_final_) were measured, and the ingestion rate was calculated as *I* = (*D*_initial_ − D_final_)/(*t* − *N*_rot_), where *t* accounts for the time in hours and *N*_rot_ is the number of rotifer individuals, for each phytoplankton species. We transformed the ingestion rates into clearance rates to obtain the volume in μL that was cleared by an individual rotifer per hour by calculating *C* = (*I*/*D*_initial_) × 10^3^.

### 2.3 Community Experiment

To investigate how initial trait distribution affects community structure and dynamics, we used six experimental phytoplankton communities (Table 1). To assemble communities, we manipulated their initial CWV and CWM by using five phytoplankton species for each community. We first plotted maximum growth rate and clearance rate as two‐dimensional trait space. Next, we calculated the smallest polygon containing all points (i.e., convex hull; Cornwell, Schwilk, and Ackerly 2006; Mammola and Cardoso 2020) as a proxy for the phytoplankton communities' initial trait distribution within the trait space for each of the communities. Communities were set up in 100 mL of Woods Hole growth medium, with each species starting from 5 × 10^3^ cells mL^−1^, following the same procedure of preparing the cultures as described above. We started the communities without and with the predator (0.5 ind mL^−1^) with *n* = 5 replicates per treatment. We sampled and diluted each flask every 4 days by replacing 10% of the culture with fresh medium. We used the removed culture for sampling. For phytoplankton, a subsample was fixed (0.01% paraformaldehyde and 0.1% glutaraldehyde final concentration) and stored at 4°C in the dark until microscopic analysis. Phytoplankton cells were classified and enumerated using a hemocytometer (Neubauer chamber) under a light‐microscope. For predator densities, we counted the rest of the sample immediately after sampling using a stereoscope.

**TABLE 1:** Replication statement.

Scale of inference	Scale at which the factor of interest is applied	Number of replicates at the appropriate scale
Trait measurements Population	13 prey species	Three replicates for growth rate measurement Five replicates for clearance rate measurement
2 Community experiment Community Population	Six different microcosm communities	Five replicates of each of the six communities

### 2.4 Statistical Analysis

All statistical analyses were performed using the software Rstudio (R version 4.2.2, R Core Team 2022). To assess significant differences between maximum growth rates, we used ANOVA using the *aov* function, and for the assessment of clearance rates of the phytoplankton species, as the data was not normally distributed, we used Kruskal‐Wallis with the *kruskal.test*, both from the “stats” R package. We calculated the trait area as the smallest polygon containing all points as a proxy for the phytoplankton communities' initial and final trait distributions using the function *chull* and *CHullArea* from the “GeoRange” R package (Developer 2017). As we used the mean trait values of each species to obtain the points for the communities, values were similar across replicates for each community. Therefore, we were not able to compare initial and final trait distributions statistically. To test for the changes and effects of CWV and CWM, we used the *weighted.mean* function from the “stats” R packages to calculate CWM and the *wtd.var* from the “Hmisc” R package (Harrell 2023) for CWV. We used evenness to estimate diversity change over time for all communities. We calculated evenness *E* as Shannon Equitability Index using the following equations, where *p*_*i*_ is the proportion of the species *i* and *H*_max_ the maximum diversity of the communities:

(2) $$H=-\Sigma {p_{i}}^{*}\;\ln\left(p_{i}\right)$$

(3) $$E=H/H_{\max}$$

After testing for normality using the *shapiro_test* function from the “rstatix” R package (Kassambara 2023), we computed generalized linear models (GLM) using the *glm* function with *Gamma* family from the “stats” R package to estimate the effects and interactions of initial trait distribution, diversity, CWV, and CWM as well as phytoplankton and predator density. To detect differences in CWV and CWM between days, we used estimated marginal means using the *emmeans* function from the “emmeans” R package (Lenth 2023) and adjusted p‐values using the “Tukey” method to correct for multiple testing. All significances were obtained using the *Anova* function from the “rstatix” R package, and model fits were inspected visually using the *allEffects* function from the “effects” R package (Fox 2003). Plots were computed using the *ggplot* function with associated subfunctions from the “ggplot2” R package (Wickham and Sievert 2016).

## 3 Results

### 3.1 Traits and Trait Space

**FIGURE 1:**
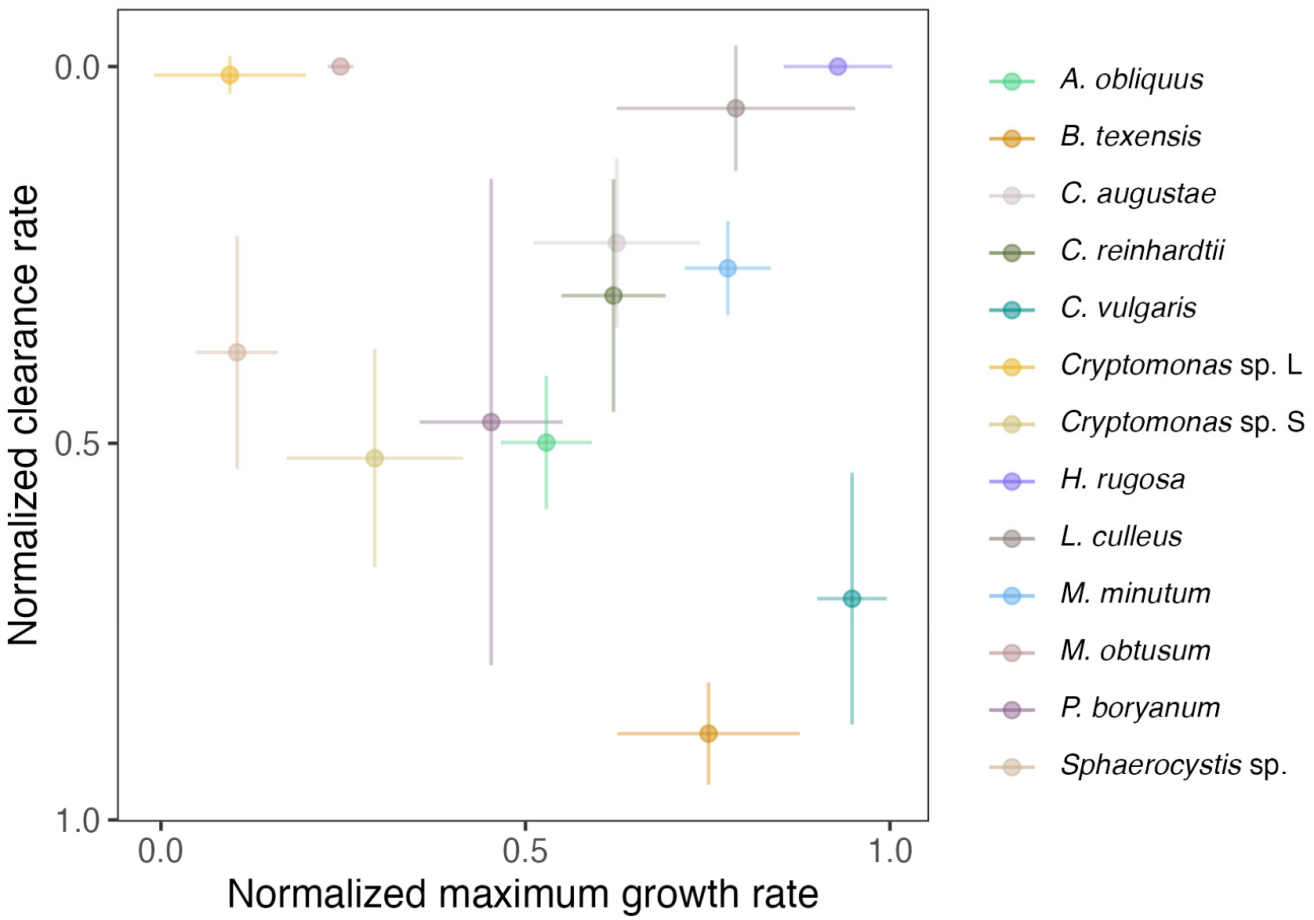
Defense–competition trait space for 13 phytoplankton species (colors; see Materials and Methods for more details). Shown are mean traits (dots) with standard deviations from independent measurements (*n* = 3 for maximum growth rate and *n* = 5 for clearance rate; vertical and horizontal lines, Table S4). Low values in clearance rate represent high defense against grazing of the predator, whereas high values account for weakly defended phytoplankton species (Note that the orientation of the y‐axis is reversed, so that high defense levels are at the top of the scale). Low maximum growth rates suggest low competitive abilities, while high values suggest high competitiveness of the species.

**FIGURE 2:**
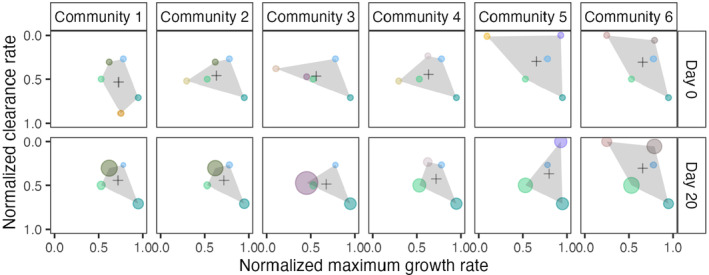
Defense–competition trait space for six experimental phytoplankton communities at the start of the experiment (top row) and after 20 days (bottom row). Mean trait values of replicates for each phytoplankton species (dots; for colors and species, see Figure 1) are shown, where the sizes of the dot represent the phytoplankton species frequency across replicates (*n* = 5). The gray area shows the smallest polygon (i.e., convex hull) containing all points, which we used as a proxy for the phytoplankton communities' initial trait distributions. The black + represents the weighted community mean value for the two traits. For the initial trait distribution at day 0, we composed communities in a way that the area of the polygon increased from community 1 to community 6, mainly through an increase in the mean defense. All species were started at equal frequencies at day 0.

We composed six communities, each consisting of five phytoplankton species, that differed significantly in their maximum growth rate (ANOVA, *F* = 29.455, df = 12, *p*‐value = 4.527 × 10^−12^) and predator clearance rate (Kruskal‐Wallis, *χ* ^2^ = 53.202, df = 12, *p*‐value = 3.793 × 10^−7^; Figure 1; Table S2). Although all communities had at least three out of five species in common, the communities differed in the area they covered in a defense–growth trait space and thus their initial trait distribution (Figure 2). The area covered by the communities ranged from 0.139 to 0.352 (Table S3). CWV for defense ranged from 0.021 to 0.076 and for growth from 0.021 to 0.101. CWM for clearance rate ranged from to 0.532 (high clearance rate = low defense) to 0.297 (low clearance rate = high defense) and for maximum growth rate from 0.562 to 0.725. Note that we normalized the trait values for equal weights of the traits. For non‐normalized trait values, see Figures S1 and S2, Tables S2 and S3.

We tracked phytoplankton community composition over 20 days. In the treatments without the predator, communities were dominated by a single species at day 20; *C. reinhardtii* in communities 1 and 2, and *M. minutum* in the other communities where *C. reinhardtii* was not present, (data not shown). As there was almost no diversity maintained in these communities and the results are in accordance with coexistence theory based on a limiting number of resources (MacArthur and Levins 1967), we did not further analyze samples from this treatment. In contrast, higher species diversity was maintained in the communities that were started with the predator. In communities 1–5, four out of five species were present at day 20. In community 6, where the initial distribution of traits was high, all species were present at the end of the experiment. Comparing the initial trait distributions (area covered in trait space) of the communities at day 0 (Figure 2) with the final trait distributions at day 20 for the communities that started with the predator (Figure 2), we observed a decrease in trait variation for five of the six communities, which was due to the loss of species. Overall, the communities differed significantly in their final diversity calculated as Shannon evenness *E* (GLM, community: *F* = 26.142, df = 5, *p*‐value = 5.587 × 10^−9^; Figure 4).

### 3.2 Changes in CWV Over Time

**FIGURE 3:**
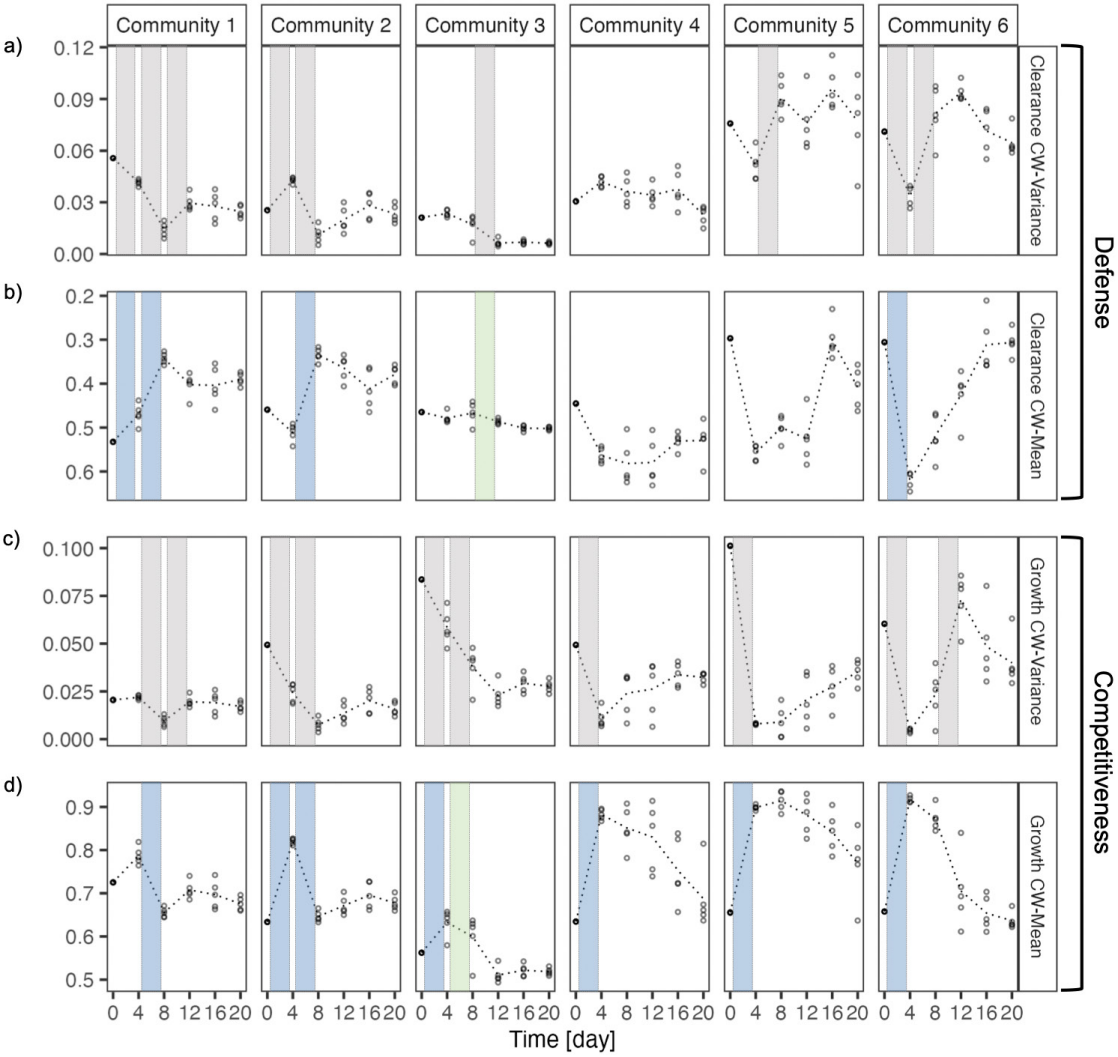
Community‐weighted variance (CWV) and community‐weighted mean (CWM) over time for six communities. (a, b) for clearance rate and (c, d) for maximum growth rate. Open points represent replicates (*n* = 5). Dotted lines show the mean of the replicates. Significant changes between days in CWV are represented by gray bars. Colored bars in CWM depict the type of selection: Blue bars represent directional selection, whereas green bars show stabilizing selection. (Note that the y‐axis for clearance CWM is reversed, so that high defense levels are at the top of the axis). For all significant changes between days for CWM, see Tables S6–S17.

CWV for the clearance rate changed over time depending on the initial trait distribution and was significantly higher for low initial trait distributions (GLM, day: *F* = 0.879, df = 1, *p*‐value < 0.35; trait area: *F* = 146.425, df = 1, *p*‐value < 2.2 × 10^−16^; day × trait area: *F* = 10.565, df = 1, *p*‐value = 0.001). For maximum growth rate, CWV decreased significantly with increasing initial trait distribution and increased over time (GLM, day: *F* = 19.479, df = 1, *p*‐value = 1.775 × 10^−5^; trait area: *F* = 6.162, df = 1, *p*‐value = 0.014; day × trait area: *F* = 0.198, df = 1, *p*‐value = 0.657). We tested for changes in CWV between days for each community and found significant differences between days, and the differences varied across communities. More specifically for clearance rate, we observed significant changes in CWV around day 8. Communities 1 and 2 showed a significant decrease from day 4 to day 8, while the decrease in community 3 occurred between days 8 and 12. Community 5 only showed a significant increase between day 4 and day 8. For community 6, we noted a decrease at the start of the experiment (Figure 3a, Tables S6–S11). As for maximum growth rate, CWV decreased significantly between day 4 and day 8, followed by an increase from day 8 to day 12 for community 1. While in communities 2 and 3, we observed decreases both after day 0 and day 4. For communities 4–6, all showed significant decreases in CWV at the start of the experiment (Figure 3c, Tables S12–S17).

### 3.3 Changes in CWM Over Time

CWM for clearance rate decreased significantly over time (i.e., increase in defense) and depended on the initial trait distribution with lower defense for lower initial trait distributions (GLM, day: *F* = 5.699, df = 1, *p*‐value = 0.018; trait area: *F* = 3.897, df = 1, *p*‐value < 0.05; day × trait area: *F* = 0.122, df = 1, *p*‐value = 0.727). CWM for maximum growth rate also varied over time and was a function of the initial trait distribution with higher CWM for higher initial trait distribution (GLM, day: *F* = 4.123, df = 1, *p*‐value = 0.044; trait area: *F* = 40.322, df = 1, *p*‐value = 1.795 × 10^−9^; day × trait area: *F* = 1.478, df = 1, *p*‐value = 0.226). Testing for differences between days: for CWM of clearance rate, we found significant changes of CWM from day 4 to day 8 for communities 1 and 2, while community 6 showed a significant increase in CWM at the start of the experiment (Figure 3b, Tables S6–S11). However, for CWM of maximum growth rate, we observed significant decreases between day 4 and day 8 for communities 1 and 2, whereas community 3 showed significant changes at the start of the experiment. For all other communities 4–6, CWM significantly increased at the start of the experiment (Figure 3d, Tables S12–S17).

### 3.4 Changes in Species Diversity Over Time

**FIGURE 4:**
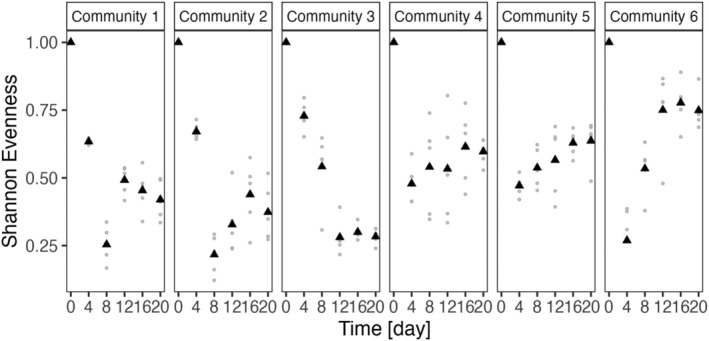
Shannon evenness *E* index for six phytoplankton communities over time. Triangles are mean values of the replicated communities (*n* = 5), and the small gray dots represent values of each replicate. Evenness decreased in all communities compared as a function of initial trait distribution (area in defense–competition trait space, Figure 2). In communities with higher initial trait distribution, final evenness was higher compared to other communities.

Over time, evenness *E* of the phytoplankton communities decreased for all six communities, and the decrease significantly depended on the initial trait distribution (GLM, day: *F* = 64.877, df = 1, *p*‐value = 1.187 × 10^−13^, area: *F* = 8.993, df = 1, *p*‐value = 0.003; area × day: *F* = 19.652, df = 1, *p*‐value = 1.636 × 10^−5^; Figure 4) and was the strongest for the communities with low initial trait distribution. Changes in evenness *E* over time correlated with changes in CWV for clearance rate and maximum growth rate, with decreases in *E* as a function of increasing CWV for all communities except for community 6, where increasing CWV resulted in increasing *E* (GLM, maximum growth rate: *F* = 106.994, df = 1, *p*‐value < 2.2 × 10^−16^; clearance rate: *F* = 17.773, df = 1, *p*‐value = 3.984 × 10^−5^; maximum growth rate*clearance rate: *F* = 50.823, df = 1, *p*‐value = 2.551 × 10^−11^). For CWM, *E* only changed significantly with CWM for clearance rate with decreasing *E* when CWM increased (GLM, maximum growth rate: *F* = 0.0034, df = 1, *p*‐value = 0.953; clearance rate: *F* = 4.105, df = 1, *p*‐value = 0.044; maximum growth rate × clearance rate: *F* = 1.353, df = 1, *p*‐value = 0.246).

### 3.5 Phytoplankton and Predator Dynamics

**FIGURE 5:**
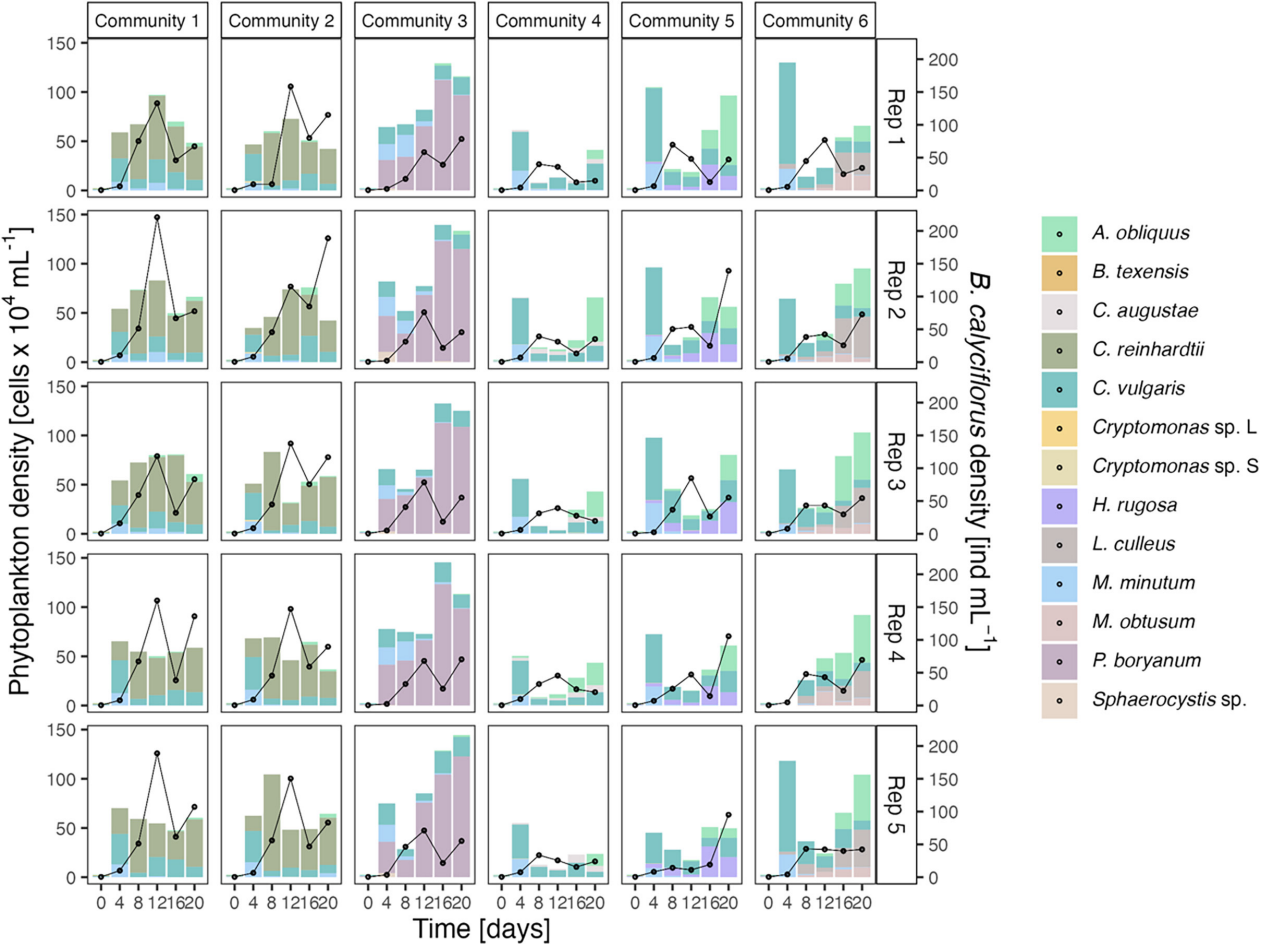
Densities of phytoplankton and *B. calyciflorus* populations over time. Shown are phytoplankton densities per species (colored bars) and *B. calyciflorus* densities (black line) for all six communities with the respective replicates as rows (for better visualization, we present the replicates as individual panels). Predator population changed significantly over time and depended on initial trait distributions (for details, see main text).

Phytoplankton densities (i.e., across all phytoplankton species) changed significantly over time, but the changes showed no dependence on the initial trait distribution (GLM, day: *F* = 34.917, df = 1, *p*‐value = 1.756 × 10^−8^; trait area: *F* = 1.141, df = 1, *p*‐value = 0.287; trait area × day: *F* = 0.017, df = 1, *p*‐value = 0.897; Figure 5). Phytoplankton densities over time were, however, significantly affected by changes in CWV and CWM for the maximum growth rate. Neither CWV or CWM for clearance rate or predator density alone nor the interaction between CWM for both maximum growth rate and clearance rate and predator density had an effect on phytoplankton density (GLM, maximum growth rate (CWV): *F* = 21.437, df = 1, *p*‐value = 7.205 × 10^−6^; maximum growth rate (CWM): *F* = 18.350, df = 1, *p*‐value = 3.060 × 10^−5^; only significant results shown; for full results, see Table S18). Testing for the effects of the initial trait distribution on the temporal dynamics of the predator population, we found that predator densities changed significantly over time and as a function of the initial trait distribution in the phytoplankton community (GLM, day: *F* = 84.654, df = 1, *p*‐value < 2 × 10^−16^; trait area: *F* = 5.485, df = 1, *p*‐value = 0.020; trait area × day: *F* = 0.802, df = 1, *p*‐value = 0.372), with the overall lower predator densities in communities with higher initial trait distribution.

We tested for the effect of both predator density and initial trait distribution on CWV and CWM. For clearance rate, both CWV and CWM were significantly dependent on both predator density and initial trait distribution, with CWV and CWM increasing at higher predator densities and higher initial trait distributions (for CWV: GLM, predator density: *F* = 1.027, df = 1, *p*‐value = 0.312; trait area: *F* = 273.127; df = 1, *p*‐value < 2 × 10^−16^; predator density × trait area: *F* = 5.372, df = 1, *p*‐value = 0.0216; for CWM: GLM, predator density: *F* = 14.879 df = 1, *p*‐value < 0.001; trait area: *F* = 6.702; df = 1, *p*‐value = 0.010; predator density × trait area: *F* = 6.888, df = 1, *p*‐value = 0.009). However, for maximum growth rate, CWV only showed a dependence on predator density, with CWV decreasing at higher predator densities but not on the initial trait distribution (GLM, predator density: *F* = 17.107, df = 1, *p*‐value = 5.477 × 10^−5^; trait area: *F* = 3.011; df = 1, *p*‐value = 0.084; predator density × trait area: *F* = 0.0000, df = 1, *p*‐value = 0.995). However, the CWM for the maximum growth rate only changed as a function of initial trait distribution, with an increase in CWM for communities with higher initial trait distribution (GLM, predator density: *F* = 0.651, df = 1, *p*‐value = 0.421; trait area: *F* = 39.237; df = 1, *p*‐value = 2.822 × 10^−9^; predator density × trait area: *F* = 0.337, df = 1, *p*‐value = 0.562).

### 3.6 Community Dynamics

We finally tested for the stability of the phytoplankton community and the predator population measured as the coefficient of variation (CV) depending on the initial trait distribution. CV changed significantly for predator populations with higher CV for communities with lower initial trait distribution (GLM, trait area: *F* = 4.218; df = 1, *p*‐value = 0.049), but CV did not change for the phytoplankton community (GLM, trait area: *F* = 3.891; df = 1, *p*‐value = 0.059).

## 4 Discussion

Previous theoretical and empirical work have shown that predation can favor the coexistence or persistence of prey species if predators prevent the extinction of more defended but less competitive species by increasing the number of niches available and thus affect community assembly (Chase et al. 2002; Chesson 2000; Hiltunen and Laakso 2013). Predator–prey interactions often lead to cyclic dynamics and thus to continuous changes in selection for competition and defense against predation. Selection for species diversity and traits can thus change over time and on short timescales, also cyclic dynamics have been shown to promote higher diversity (Chase et al. 2002; Chesson 2000; Chesson and Kuang 2008; Hiltunen et al. 2012; Klauschies, Vasseur, and Gaedke 2016). Because species diversity and trait variation in the prey community determine the dynamics of the predator population or community, there is a potential for feedback between community assembly and predator dynamics, tied via selection. The species pool and the trait variation that can result from this should thus play a key role in this feedback. We tested how changes in species diversity and trait variation over time were related in communities that differed in their species composition and initial trait distributions for competitiveness, measured as maximum growth rate and clearance rate as a mean for defense against predation. We examined how selection on species diversity and those traits correlated with changes in predator density over time. We found that predation promoted the persistence of species and thus maintained higher species diversity, but that species diversity changed over time depending on the initial trait distribution and the predator densities. This was due to predator selection over time and was dependent on the initial trait distribution as changes in CWV and CWM varied with predator densities and were dependent on the initial trait distribution.

Decreases in CWV in combination with changes in CWM resulted from directional selection on traits. The decrease in CWV without a change in CWM was interpreted as stabilizing selection and the increases in CWV as greater niche availability. More specifically, the changes in species diversity were attributed to changes in selection over time, as we observed that diversity, measured as evenness *E*, correlated with changes in CWV for defense and competitiveness. We found strong reductions in CWV in both traits at lower but increasing predator densities, suggesting selection. However, the dynamics differed depending on the initial trait distribution in the communities. Communities with lower initial trait distributions (communities 1 and 2) had a strong decrease in CWV for defense and competitiveness around day 8, and this decrease coincided with an increase in mean defense, but a decrease in mean competitiveness, suggesting directional selection for higher levels of defense with lower competitive abilities in the communities. For community 3, we also observed a decrease in CWV for defense and competitiveness around day 8, but no change in CWM for defense, suggesting stabilizing selection. In communities 1–3, competitiveness was under directional selection. In community 4, we found early directional selection for competitiveness, as we observed a decrease in CWV and an increase in CWM at the start of the experiment. For community 5, with the highest initial trait distribution, we also observed a decrease in CWV for competitiveness in the first part of the experiment. These changes correlated with an increase in CWM for competitiveness, suggesting directional selection for higher competitive ability when predator densities are low. In community 6, where all species were present at the end of the experiment, defense and competitiveness were under directional selection. In communities 1 and 6, after the initial period of selection, we observed an increase in CWV around day 8 for both defense and competitiveness. In community 5, we only found an increase in CWV for defense. Following DeMalach, Ke, and Fukami (2022), we interpret this as evidence for a higher availability of niches. For disruptive selection, we would expect increases in CWV but no change in CWM (Rolhauser and Pucheta 2017). However, predictions about CWM are less clear for niche availability as we observed both significant changes in CWM and no changes in CWM, e.g., in community 6, the significant increase in CWV for defense coincided with no significant change in CWM for defense. While, for competitiveness, the increase in CWV was accompanied by a significant change in CWM.

Negative correlations between traits and trade‐offs are common and have been observed in phytoplankton (Cadier et al. 2019; Ehrlich, Kath, and Gaedke 2020; Fussmann et al. 2005), making it somewhat more difficult to interpret which trait might be under selection in our experiment. We often find that selection on defense and competitiveness changes together, and that selection favors higher defense and lower competitiveness, indicating a classic trade‐off scenario (communities 1, 2, 5, 6; Kasada, Yamamichi, and Yoshida 2014; Pančić and Kiørboe 2018; Yoshida et al. 2003; Yoshida, Hairston, and Ellner 2004). However, we have also found cases where stabilizing selection for defense is favored, but directional selection for higher competitiveness (community 3), suggesting that trade‐offs were not always important here. This observation is counterintuitive and contrary to the findings of other studies (Pančić and Kiørboe 2018; Yoshida, Hairston, and Ellner 2004) but is consistent with studies that have found that trade‐offs may be context‐dependent (Edwards, Klausmeier, and Litchman 2011; Meaden, Paszkiewicz, and Koskella 2015). Although other studies have previously shown that maximum growth rate and clearance rate are good proxies to describe competition and defense against consumption, it is possible that other traits underlying competition and defense would show the trade‐off in all communities and might as well explain why the persisting species in community 5 are not all outcompeted by the species with a high maximum growth rate and high levels of defense against predation. This warrants a more careful interpretation of correlated trait changes within communities and increased efforts to identify the traits under selection and their link to performance (Laughlin 2014; Laughlin and Messier 2015; Réveillon and Becks 2024). Furthermore, it indicates the need for more research using more and/or other traits to investigate the relationship of initial trait distribution and species diversity.

The framework presented by DeMalach, Ke, and Fukami (2022) assumes that the species pool is shared across communities and that trait differences reflect competitive hierarchy instead of niche partitioning and that selection is identified by comparing to the null model based on the trait distribution of all species in the pool. Our application here differs from these assumptions and the comparison to the null model in several ways. First, we identified selection based on the comparison between time points rather than the null model. We did this because we knew environmental changes over time (i.e., predator densities) and could attribute changes in diversity and CWV to predator selection: Higher predator densities increase selection strength for defense while lower predator densities lead to stronger selection for competitiveness. In line with this prediction, we found that changes in CWV correlated with predator density.

Second, we manipulated the species pool in the communities we compared. We did this by replacing one or two species to demonstrate that community assembly and dynamics depended on the initial trait distribution (i.e., the standing trait variation) and the effect of selection. This allowed us to test the prediction that species diversity decreases with increasing strength of selection because species with greater fitness differences are excluded more quickly (Vellend 2016) and because stronger selection leads to faster shifts in traits (DeMalach, Ke, and Fukami 2022; Li et al. 2014). Here, we focused on differences in defense and competitiveness and their role in determining fitness. We found that diversity decreased depending on the strength of selection. In communities where predator densities were high (i.e., stronger selection) species diversity was lower compared to those communities where selection was weaker. Furthermore, we were able to show that strong directional selection favored species with extreme trait values, whereas strong stabilizing selection favored species with intermediate trait values.

Finally, we did not assume a competitive hierarchy but rather that niche availability changed with predator density. This allows us to consider two (correlated) traits that contribute to fitness and for the dynamic interactions and potential feedback between predator density and trait distributions in the prey community (Koch et al. 2014; Yoshida, Hairston, and Ellner 2004). Indeed, we found that differences in initial trait distribution and subsequent trait shifts led to differences in predator population stability, with predator populations being more stable with higher initial trait distribution and CWM in defense.

Overall, by manipulating the initial species pool, thus the trait variation in the prey community that determined the dynamics of the predator population, which in return resulted in changes in species diversity as well as CWV and CWM depending on the initial trait distribution, we showed how species diversity, trait variation, and population dynamics might interact. This study is a first test of the framework proposed by DeMalach, Ke, and Fukami (2022) with controlled laboratory communities. We considered only a small number of phytoplankton species for each of our experimental communities as well as only one predator species, which does not reflect natural communities. Using this approach, we were able to obtain a more mechanistic understanding of the underlying mechanisms. By only manipulating initial trait distributions and thus species compositions, while keeping all other variables constant, we gained a first insight into the link between predator–prey dynamics and ecological selection under the light of the abovementioned framework. Adding more species and traits to the system and/or modifying additional variables, e.g., changes in temperature, or resource availability, could be an important next step. However, additions and modifications should be chosen carefully, as disentangling relationships and mechanisms can be challenging with more possible interacting variables. We support the suggestion of DeMalach and colleagues that examining the relationship between species diversity and trait variation through ecological selection as a common framework can provide an important mechanistic understanding of community assembly and help identify patterns of diversity.

## Author Contributions

**Phuong‐Anh Vu:** conceptualization (lead), data curation (lead), formal analysis (equal), investigation (lead), methodology (equal), project administration (lead), validation (supporting), visualization (lead), writing – original draft (lead), writing – review and editing (equal). **Lutz Becks:** conceptualization (supporting), data curation (supporting), formal analysis (equal), funding acquisition (lead), investigation (supporting), methodology (equal), project administration (supporting), resources (lead), supervision (lead), validation (lead), visualization (supporting), writing – original draft (supporting), writing – review and editing (equal).

## Conflicts of Interest

The authors declare no conflicts of interest.

## Statement on Inclusion

Our study was based on experimental communities to investigate the feedback between species diversity, traits, and population dynamics. For our communities, we tried to use species that were either isolated from or occur in the local lake. For our analysis, we used a framework proposed by DeMalach, Ke, and Fukami (2022). We recognize that more could have been done to engage in intellectual exchange and collaboration with these authors and will try to address these caveats for future work.

## Supporting information


Data S1.

## Data Availability

The data presented in this study can be found in the public data archive Dryad doi:10.5061/dryad.pvmcvdnvt.
