# Supplementary material for: Community Trait Variation Drives Selection on Species Diversity Through Feedback With Predator Density
Source: Ecol Evol. 2024 Oct 23;14(10):e70477. doi: 10.1002/ece3.70477 (PMC11499210; doi:10.1002/ece3.70477)
Supplement: Supplementary file 1 — Data S1. [file ECE3-14-e70477-s001.pdf]

Supplementary Material:

Community trait variation drives selection on species diversity through feedback with predator density

Community trait variation drives selection on species diversity through feedback with predator density

Table of content

Figures

Figure S1: Non-normalized defense-competition trait space. 4

Figure S2: Non-normalized defense-competition trait space for six experimental phytoplankton communities. 5

Tables

Table S1: Species names with respective species culture identities. 3

Table S2: Non-normalized maximum growth rate and clearance rate. 6

Table S3: Non-normalized initial and final trait distributions calculated as area, community-weighted variance and community-weighted mean. 6

Table S4: Normalized maximum growth rate and clearance rate. 7

Table S5: Normalized initial and final trait distributions calculated as area, community-weighted variance and community-weighted mean. 7

Table S6: Analysis of Deviance and Estimated Marginal Means Table extracted from R for CWV and CWM for clearance rate of community 1. 8

Table S7: Analysis of Deviance and Estimated Marginal Means Table extracted from R for CWV and CWM for clearance rate of community 2. 10

Table S8: Analysis of Deviance and Estimated Marginal Means Table extracted from R for CWV and CWM for clearance rate of community 3. 12

Table S9: Analysis of Deviance and Estimated Marginal Means Table extracted from R for CWV and CWM for clearance rate of community 4. 14

Table S10: Analysis of Deviance and Estimated Marginal Means Table extracted from R for CWV and CWM for clearance rate of community 5. 16

Table S11: Analysis of Deviance and Estimated Marginal Means Table extracted from R for CWV and CWM for clearance rate of community 6. 18

Table S12: Analysis of Deviance and Estimated Marginal Means Table extracted from R for CWV and CWM for maximum growth rate of community 1. 20

Table S13: Analysis of Deviance and Estimated Marginal Means Table extracted from R for CWV and CWM for maximum growth rate of community 2. 22

Table S14: Analysis of Deviance and Estimated Marginal Means Table extracted from R for CWV and CWM for maximum growth rate of community 3. 24

Table S15: Analysis of Deviance and Estimated Marginal Means Table extracted from R for CWV and CWM for maximum growth rate of community 4. 26

Table S16: Analysis of Deviance and Estimated Marginal Means Table extracted from R for CWV and CWM for maximum growth rate of community 5. 28

Table S17: Analysis of Deviance and Estimated Marginal Means Table extracted f rom R for CWV and CWM for maximum growth rate of community 6. 30

Table S18: Analysis of deviance table for the effect of CWV and CWM for defense and growth and rotifer densities on phytoplankton densities. 32

Table S1: Species names with respective species culture identities.

Species	Culture identity
Acutodesmus obliquus (formerly known as *Scenedesmus obliquus*)	SAG 276-3a
Chlorella vulgaris	SAG211-11b
Pediastrum boryanum	Isolated from Lake Constance
Monoraphidium minutum	SAG 243-1
Monoraphidium obtusum	SAG 55.81
Sphaerocystis sp.	Isolated from Lake Constance
Chlamydomonas reinhardtii	SAG 11-32b
Borodinellopsis texensis	SAG 17.95
Chloromonas augustae	SAG 13.89
Heterochlamydomonas rugosa	SAG 45.86
Lobochlamys culleus	SAG 19.72
Cryptomonas sp. (S)	SAG 979-8
Cryptomonas sp. (L)	SAG 26.80

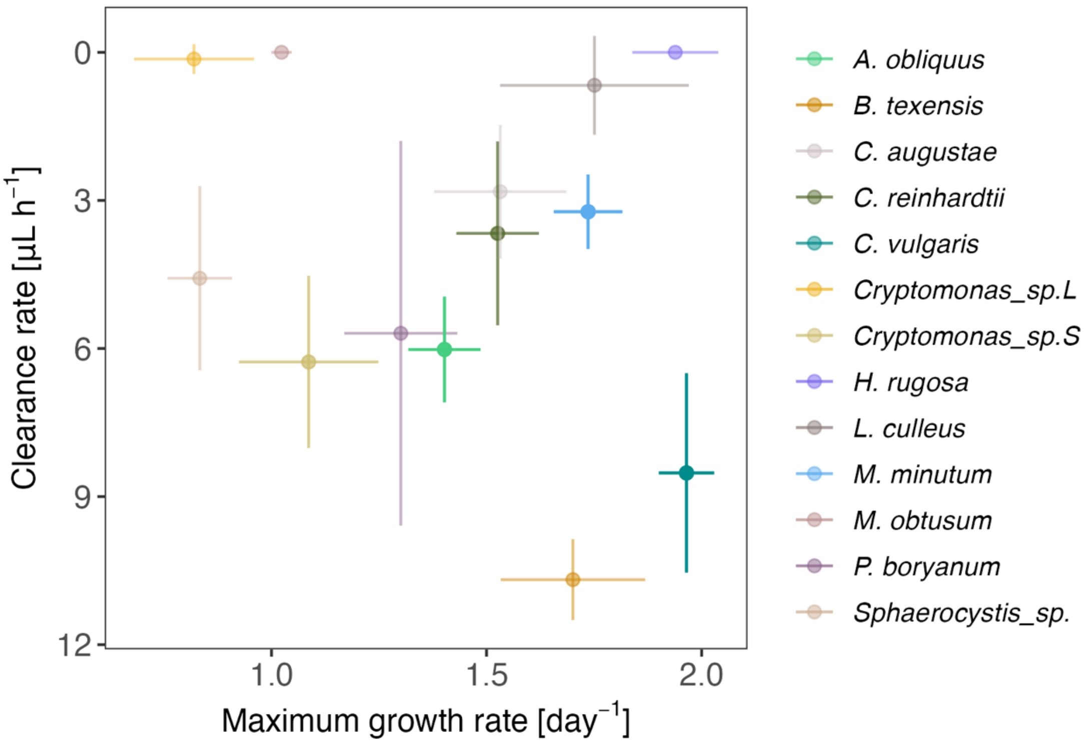

Figure S1: Non-normalized defense-competition trait space for 13 phytoplankton species (colors; see main text for more details). Shown are mean traits (dots) with standard deviations from independent measurements (n = 3 for maximum growth rate and n = 5 for clearance rate; vertical and horizontal lines, Table S2). Low values in clearance rate represent high defense against grazing of the predator, whereas high values account for weakly defended phytoplankton species (Note that the orientation of the y-axis is reversed, so that high defense levels are at the top of the scale). Low maximum growth rates suggest low competitive abilities, while high values suggest high competitiveness of the species.

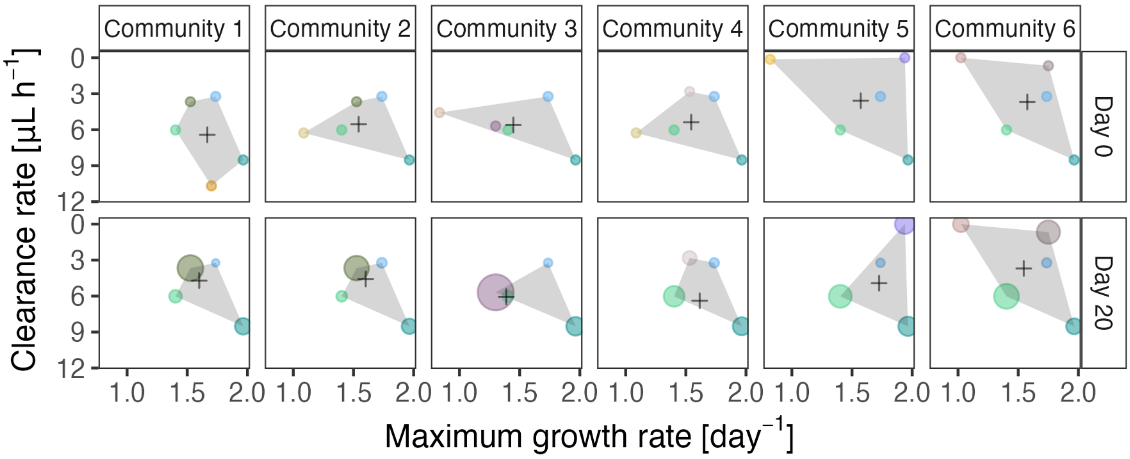

Figure S2: Non-normalized defense-competition trait space for six experimental phytoplankton communities at the start of the experiment (top row) and after 20 days (bottom row). Mean trait values of replicates for each phytoplankton species (dots, for colors see Fig. S1) are shown, where the sizes of the dot represent the phytoplankton species frequency across replicates (n = 5). The grey area (see Table S3 and S4 for specific values) shows the smallest polygon (i.e., convex hull) containing all points, which we used as a proxy for the phytoplankton communities' initial trait distributions. The black + represents the weighted community mean value for the two traits. For the initial trait distribution at day 0, we composed communities in a way that the area of the polygon increased from community 1 to community 6, mainly through an increase in the mean defense. All species were started at equal frequencies at day 0

Table S2: Non-normalized maximum growth rate as competitive ability and clearance rate as defense against predation for 13 phytoplankton species with respective standard deviations (SD). Values are means of replicates (n=3 for growth rate and n=5 for clearance rate).

Species	Growth rate (day^{-1})	Growth SD	Clearance rate ($\mu L\ h^{-1}$)	Clearance SD
A. obliquus	1.402	0.084	6.020	1.07
B. texensis	1.701	0.168	10.683	0.820
C. augustae	1.532	0.153	2.822	1.353
C. reinhardtii	1.536	0.096	3.668	1.862
C. vulgaris	1.965	0.064	8.521	2.01
Cryptomonas sp. (L)	0.82	0.14	0.136	0.302
Cryptomonas sp. (S)	1.086	0.162	6.272	1.744
H. rugosa	1.939	0.100	0.000	0.000
L. culleus	1.751	0.219	0.668	1.000
M. minutum	1.736	0.079	3.229	0.753
M. obtusum	1.023	0.024	0.000	0.000
P. boryanum	1.301	0.131	5.692	3.896
Sphaerocystis sp.	0.833	0.075	4.578	1.864

Table S3: Non-normalized initial and final trait distributions calculated as area (i.e., the smallest polygon containing all points) for six phytoplankton communities, as well as community weighted variance and community weighted mean of clearance rate and growth rate for initial and final communities

Community	Area Initial	Area Final	CWV CR Initial	CWV CR Final	CWM CR Initial	CWM CR Final	CWV GR Initial	CWV GR Final	CWM GR Initial	CWM GR Final
1	2.361	1.423	8.100	3.586	6.423	4.709	0.037	0.030	1.666	1.599
2	2.244	1.423	3.699	3.396	5.542	4.574	0.089	0.028	1.543	1.601
3	2.543	1.434	3.076	0.912	5.608	6.058	0.150	0.05	1.447	1.389
4	2.51	1.555	4.447	3.283	5.373	6.388	0.089	0.058	1.554	1.615
5	5.696	2.364	11.033	11.218	3.581	4.929	0.182	0.063	1.572	1.724
6	4.004	4.004	10.344	9.414	3.688	3.695	0.109	0.072	1.575	1.547

Table S4: Normalized maximum growth rate as competitive ability and clearance rate as defense against predation for 13 phytoplankton species with respective standard deviations (SD). Values are means of replicates (n=3 for growth rate and n=5 for clearance rate).

Species	Normalized Growth rate	Growth SD	Normalized Clearance rate	Clearance SD
A. obliquus	0.529	0.062	0.499	0.089
B. texensis	0.751	0.125	0.885	0.068
C. augustae	0.625	0.114	0.234	0.112
C. reinhardtii	0.621	0.071	0.304	0.154
C. vulgaris	0.948	0.048	0.706	0.167
Cryptomonas sp. (L)	0.094	0.104	0.011	0.025
Cryptomonas sp. (S)	0.293	0.120	0.52	0.145
H. rugosa	0.929	0.075	0.000	0.000
L. culleus	0.788	0.163	0.055	0.083
M. minutum	0.778	0.059	0.268	0.062
M. obtusum	0.246	0.018	0.000	0.000
P. boryanum	0.453	0.098	0.472	0.323
Sphaerocystis sp.	0.104	0.056	0.379	0.155

Table S5: Normalized initial and final trait distributions calculated as area (i.e., the smallest polygon containing all points) for six phytoplankton communities, as well as community weighted variance (CWV) and community weighted mean (CWM) of clearance rate (CR) and growth rate (GR) for initial and final communities

Community	Area Initial	Area Final	CWV CR Initial	CWV CR Final	CWM CR Initial	CWM CR Final	CWV GR Initial	CWV GR Final	CWM GR Initial	CWM GR Final
1	0.146	0.088	0.056	0.025	0.532	0.390	0.021	0.017	0.725	0.676
2	0.139	0.088	0.024	0.023	0.459	0.379	0.049	0.016	0.634	0.677
3	0.157	0.089	0.021	0.006	0.465	0.502	0.084	0.028	0.562	0.518
4	0.155	0.096	0.031	0.023	0.445	0.529	0.049	0.032	0.635	0.687
5	0.352	0.146	0.076	0.077	0.297	0.409	0.101	0.035	0.655	0.769
6	0.247	0.247	0.071	0.065	0.306	0.306	0.060	0.04	0.658	0.636

Table S6: Analysis of Deviance and Estimated Marginal Means Table extracted from R for a) CWV and b) CWM for defense of community 1.

a)

	F-value	df	*p*-value
day	50.667	5	$5.646*10^{-12}$

contrast	*p*-value
day0 - day4	0.0006
day0 - day8	<.0001
day0 - day12	<.0001
day0 - day16	<.0001
day0 - day20	<.0001
day4 - day8	<.0001
day4 - day12	0.0048
day4 - day16	0.0009
day4 - day20	0.0001
day8 - day12	0.0002
day8 - day16	0.0011
day8 - day20	0.0135
day12 - day16	0.9808
day12 - day20	0.5232
day16 - day20	0.8994

b)

	F-value	df	p-value
day	39.262	5	$8.641*10^{-11}$

contrast	p-value
day0 - day4	0.0044
day0 - day8	<.0001
day0 - day12	<.0001
day0 - day16	<.0001
day0 - day20	<.0001
day4 - day8	<.0001
day4 - day12	0.0021
day4 - day16	0.0027
day4 - day20	0.0003
day8 - day12	0.0069
day8 - day16	0.0054
day8 - day20	0.0431
day12 - day16	1.0000
day12 - day20	0.9663
day16 - day20	0.9446

Table S7: Analysis of Deviance and Estimated Marginal Means Table extracted from R for a) CWV and b) CWM for defense of community 2.

a)

	F-value	df	*p*-value
day	19.546	5	$9.257*10^{-8}$

contrast	*p*-value
day0 - day4	0.0003
day0 - day8	0.0032
day0 - day12	0.5823
day0 - day16	0.9701
day0 - day20	0.9881
day4 - day8	<.0001
day4 - day12	<.0001
day4 - day16	0.0022
day4 - day20	0.0001
day8 - day12	0.1270
day8 - day16	0.0005
day8 - day20	0.0142
day12 - day16	0.1929
day12 - day20	0.9081
day16 - day20	0.7307

b)

	F-value	df	*p*-value
day	32.405	5	$6.377*10^{-10}$

contrast	*p*-value
day0 - day4	0.0392
day0 - day8	<.0001
day0 - day12	0.0001
day0 - day16	0.0714
day0 - day20	0.0006
day4 - day8	<.0001
day4 - day12	<.0001
day4 - day16	<.0001
day4 - day20	<.0001
day8 - day12	0.4735
day8 - day16	0.0010
day8 - day20	0.1048
day12 - day16	0.0720
day12 - day20	0.9385
day16 - day20	0.3694

Table S8: Analysis of Deviance and Estimated Marginal Means Table extracted from R for a) CWV and b) CWM for defense of community 3.

a)

	F-value	df	*p*-value
day	40.516	5	$6.2*10^{-11}$

contrast	*p*-value
day0 - day4	0.7545
day0 - day8	0.2227
day0 - day12	<.0001
day0 - day16	<.0001
day0 - day20	<.0001
day4 - day8	0.0134
day4 - day12	<.0001
day4 - day16	<.0001
day4 - day20	<.0001
day8 - day12	<.0001
day8 - day16	0.0001
day8 - day20	<.0001
day12 - day16	0.9997
day12 - day20	1.0000
day16 - day20	0.9994

b)

	F-value	df	*p*-value
day	9.4649	5	$4.376*10^{-5}$

contrast	*p*-value
day0 - day4	0.4642
day0 - day8	0.9998
day0 - day12	0.0756
day0 - day16	0.0007
day0 - day20	0.0006
day4 - day8	0.6247
day4 - day12	0.8914
day4 - day16	0.0551
day4 - day20	0.0445
day8 - day12	0.1278
day8 - day16	0.0014
day8 - day20	0.0011
day12 - day16	0.3795
day12 - day20	0.3287
day16 - day20	1.0000

Table S9: Analysis of Deviance and Estimated Marginal Means Table extracted from R for a) CWV and b) CWM for defense of community 4.

a)

	F-value	df	*p*-value
day	5.2618	5	0.002112

contrast	*p*-value
day0 - day4	0.1030
day0 - day8	0.7201
day0 - day12	0.9515
day0 - day16	0.5321
day0 - day20	0.4032
day4 - day8	0.7650
day4 - day12	0.4379
day4 - day16	0.9058
day4 - day20	0.0012
day8 - day12	0.9934
day8 - day16	0.9996
day8 - day20	0.0282
day12 - day16	0.9531
day12 - day20	0.0910
day16 - day20	0.0138

b)

	F-value	df	p-value
day	9.7648	5	$3.456*10^{-5}$

contrast	p-value
day0 - day4	0.0004
day0 - day8	0.0001
day0 - day12	0.0001
day0 - day16	0.0128
day0 - day20	0.0143
day4 - day8	0.9637
day4 - day12	0.9797
day4 - day16	0.7166
day4 - day20	0.6896
day8 - day12	1.0000
day8 - day16	0.2642
day8 - day20	0.2451
day12 - day16	0.3105
day12 - day20	0.2891
day16 - day20	1.0000

Table S10: Analysis of Deviance and Estimated Marginal Means Table extracted from R for a) CWV and b) CWM for defense of community 5.

a)

	F-value	df	*p*-value
day	6.0091	5	0.0009677

contrast	*p*-value
day0 - day4	0.1117
day0 - day8	0.5585
day0 - day12	1.0000
day0 - day16	0.2378
day0 - day20	1.0000
day4 - day8	0.0025
day4 - day12	0.1057
day4 - day16	0.0005
day4 - day20	0.0843
day8 - day12	0.5758
day8 - day16	0.9893
day8 - day20	0.6452
day12 - day16	0.2492
day12 - day20	1.0000
day16 - day20	0.2986

b)

	F-value	df	p-value
day	48.3	5	$9.474*10^{-12}$

contrast	p-value
day0 - day4	<.0001
day0 - day8	<.0001
day0 - day12	<.0001
day0 - day16	1.0000
day0 - day20	0.0009
day4 - day8	0.1674
day4 - day12	0.7347
day4 - day16	<.0001
day4 - day20	<.0001
day8 - day12	0.8758
day8 - day16	<.0001
day8 - day20	0.0075
day12 - day16	<.0001
day12 - day20	0.0005
day16 - day20	0.0012

Table S11: Analysis of Deviance and Estimated Marginal Means Table extracted from R for a) CWV and b) CWM for defense of community 6.

a)

	F-value	df	*p*-value
day	19.187	5	$1.587*10^{-7}$

contrast	*p*-value
day0 - day4	0.0001
day0 - day8	0.5383
day0 - day12	0.0132
day0 - day16	1.0000
day0 - day20	0.8985
day4 - day8	<.0001
day4 - day12	<.0001
day4 - day16	0.0001
day4 - day20	0.0009
day8 - day12	0.3773
day8 - day16	0.5804
day8 - day20	0.1023
day12 - day16	0.0154
day12 - day20	0.0011
day16 - day20	0.8718

b)

	F-value	df	p-value
day	41.084	5	$1.013*10^{-10}$

contrast	p-value
day0 - day4	<.0001
day0 - day8	<.0001
day0 - day12	0.0030
day0 - day16	0.9999
day0 - day20	1.0000
day4 - day8	0.0201
day4 - day12	<.0001
day4 - day16	<.0001
day4 - day20	<.0001
day8 - day12	0.0306
day8 - day16	<.0001
day8 - day20	<.0001
day12 - day16	0.0050
day12 - day20	0.0032
day16 - day20	1.0000

Table S12: Analysis of Deviance and Estimated Marginal Means Table extracted from R for a) CWV and b) CWM for competitiveness of community 1.

a)

	F-value	df	*p*-value
day	10.457	5	$2.039*10^{-5}$

contrast	*p*-value
day0 - day4	0.9790
day0 - day8	0.0001
day0 - day12	0.9966
day0 - day16	0.9750
day0 - day20	0.5126
day4 - day8	<.0001
day4 - day12	0.8396
day4 - day16	0.6994
day4 - day20	0.1755
day8 - day12	0.0003
day8 - day16	0.0006
day8 - day20	0.0073
day12 - day16	0.9998
day12 - day20	0.7943
day16 - day20	0.9084

b)

	F-value	df	p-value
day	27.909	5	$2.908*10^{-9}$

contrast	p-value
day0 - day4	0.0005
day0 - day8	0.0001
day0 - day12	0.7145
day0 - day16	0.2278
day0 - day20	0.0058
day4 - day8	<.0001
day4 - day12	<.0001
day4 - day16	<.0001
day4 - day20	<.0001
day8 - day12	0.0030
day8 - day16	0.0251
day8 - day20	0.5541
day12 - day16	0.9449
day12 - day20	0.1335
day16 - day20	0.5340

Table S13: Analysis of Deviance and Estimated Marginal Means Table extracted from R for a) CWV and b) CWM for competitiveness of community 2.

a)

	F-value	df	*p*-value
day	56.389	5	$1.759*10^{-12}$

contrast	*p*-value
day0 - day4	<.0001
day0 - day8	<.0001
day0 - day12	<.0001
day0 - day16	<.0001
day0 - day20	<.0001
day4 - day8	<.0001
day4 - day12	0.0084
day4 - day16	0.6639
day4 - day20	0.0542
day8 - day12	0.2746
day8 - day16	0.0016
day8 - day20	0.0584
day12 - day16	0.2070
day12 - day20	0.9609
day16 - day20	0.6382

b)

	F-value	df	*p*-value
day	72.064	5	$1.167*10^{-13}$

contrast	*p*-value
day0 - day4	<.0001
day0 - day8	0.8687
day0 - day12	0.0275
day0 - day16	0.0001
day0 - day20	0.0078
day4 - day8	<.0001
day4 - day12	<.0001
day4 - day16	<.0001
day4 - day20	<.0001
day8 - day12	0.2552
day8 - day16	0.0024
day8 - day20	0.0943
day12 - day16	0.2953
day12 - day20	0.9937
day16 - day20	0.6001

Table S14: Analysis of Deviance and Estimated Marginal Means Table extracted from R for a) CWV and b) CWM for competitiveness of community 3.

a)

	F-value	df	*p*-value
day	63.45	5	$4.802*10^{-13}$

contrast	*p*-value
day0 - day4	<.0001
day0 - day8	<.0001
day0 - day12	<.0001
day0 - day16	<.0001
day0 - day20	<.0001
day4 - day8	0.0006
day4 - day12	<.0001
day4 - day16	<.0001
day4 - day20	<.0001
day8 - day12	0.0175
day8 - day16	0.3436
day8 - day20	0.1798
day12 - day16	0.6632
day12 - day20	0.8715
day16 - day20	0.9986

b)

	F-value	df	p-value
day	17.075	5	$3.219*10^{-7}$

contrast	p-value
day0 - day4	0.0046
day0 - day8	0.2827
day0 - day12	0.0574
day0 - day16	0.2031
day0 - day20	0.1441
day4 - day8	0.4043
day4 - day12	<.0001
day4 - day16	<.0001
day4 - day20	<.0001
day8 - day12	0.0003
day8 - day16	0.0016
day8 - day20	0.0010
day12 - day16	0.9859
day12 - day20	0.9971
day16 - day20	1.0000

Table S15: Analysis of Deviance and Estimated Marginal Means Table extracted from R for a) CWV and b) CWM for competitiveness of community 4.

a)

	F-value	df	*p*-value
day	11.906	5	$7.231*10^{-6}$

contrast	*p*-value
day0 - day4	<.0001
day0 - day8	0.0008
day0 - day12	0.0022
day0 - day16	0.0623
day0 - day20	0.0346
day4 - day8	0.1517
day4 - day12	0.0657
day4 - day16	0.0023
day4 - day20	0.0045
day8 - day12	0.9981
day8 - day16	0.4490
day8 - day20	0.6137
day12 - day16	0.7045
day12 - day20	0.8482
day16 - day20	0.9998

b)

	F-value	df	p-value
day	14.55	1	$1.333*10^{-6}$

contrast	p-value
day0 - day4	<.0001
day0 - day8	0.0001
day0 - day12	0.0002
day0 - day16	0.0348
day0 - day20	0.6976
day4 - day8	0.9527
day4 - day12	0.7038
day4 - day16	0.0180
day4 - day20	0.0002
day8 - day12	0.9914
day8 - day16	0.1141
day8 - day20	0.0018
day12 - day16	0.3155
day12 - day20	0.0075
day16 - day20	0.4795

Table S16: Analysis of Deviance and Estimated Marginal Means Table extracted from R for a) CWV and b) CWM for competitiveness of community 5.

a)

	F-value	df	*p*-value
day	94.118	5	$5.771*10^{-15}$

contrast	*p*-value
day0 - day4	<.0001
day0 - day8	<.0001
day0 - day12	<.0001
day0 - day16	<.0001
day0 - day20	<.0001
day4 - day8	0.9999
day4 - day12	0.1549
day4 - day16	0.0095
day4 - day20	0.0002
day8 - day12	0.2217
day8 - day16	0.0153
day8 - day20	0.0003
day12 - day16	0.7871
day12 - day20	0.0820
day16 - day20	0.6276

b)

	F-value	df	*p*-value
day	25.741	5	$6.513*10^{-9}$

contrast	*p*-value
day0 - day4	<.0001
day0 - day8	<.0001
day0 - day12	<.0001
day0 - day16	<.0001
day0 - day20	0.0045
day4 - day8	0.9924
day4 - day12	0.9861
day4 - day16	0.3416
day4 - day20	0.0011
day8 - day12	0.8276
day8 - day16	0.1303
day8 - day20	0.0003
day12 - day16	0.7199
day12 - day20	0.0053
day16 - day20	0.1220

Table S17: Analysis of Deviance and Estimated Marginal Means Table extracted from R for a) CWV and b) CWM for competitiveness of community 6.

a)

	F-value	df	*p*-value
day	17.536	5	$3.543*10^{-7}$

contrast	*p*-value
day0 - day4	<.0001
day0 - day8	0.0016
day0 - day12	0.6075
day0 - day16	0.6837
day0 - day20	0.1531
day4 - day8	0.2595
day4 - day12	<.0001
day4 - day16	0.0004
day4 - day20	0.0043
day8 - day12	<.0001
day8 - day16	0.0480
day8 - day20	0.3450
day12 - day16	0.0534
day12 - day20	0.0047
day16 - day20	0.8886

b)

	F-value	df	p-value
day	40.016	5	$1.325*10^{-10}$

contrast	p-value
day0 - day4	<.0001
day0 - day8	<.0001
day0 - day12	0.4800
day0 - day16	1.0000
day0 - day20	0.9606
day4 - day8	0.5966
day4 - day12	<.0001
day4 - day16	<.0001
day4 - day20	<.0001
day8 - day12	<.0001
day8 - day16	<.0001
day8 - day20	<.0001
day12 - day16	0.4020
day12 - day20	0.1300
day16 - day20	0.9819

Table S18: Analysis of deviance table for the effect of CWV and CWM for defense and growth and rotifer densities on phytoplankton densities.

	df	*F*-value	*p*-value
g_CWV	1	21.4373	$7.205*10^{-6}$
i_CWV	1	1.6574	0.19969
rt_mean_mL	1	0.0974	0.75536
g_CWM	1	18.3500	$3.060*10^{-5}$
i_CWM	1	0.0000	0.99712
rt_mean_mL:g_CWM	1	2.0551	0.15352
rt_mean_mL:i_CWM	1	1 3.2126	0.07484
